# Supplementary material for: The Role of Grass in the Epidemiology of a Phytoplasma Disease Affecting Trees and Other Plants of the Sabana de Bogotá, Colombia
Source: Microorganisms. 2025 Apr 23;13(5):967. doi: 10.3390/microorganisms13050967 (PMC12114218; doi:10.3390/microorganisms13050967)
Supplement: Supplementary file 1 [file microorganisms-13-00967-s001.zip › microorganisms-3553787-supplementary.pdf]

Table S1. Number of weed specimens collected per species and zone

Amaranthus dubius Mart. ex Thell.	11	E, C, W, N
Baccharis latifolia (Ruiz and Pav.)	2	W
Capsella bursa-pastoris (L.) Medik	7	S, C, N
Cenchrus clandestinus (Hochst. ex Chiov.) Morrone	6	S,W,N
Chenopodium album L.	5	C, N, E
Cotula australis (Sieber ex Spreng.) Hook. F.	5	N,C,S
Cymbalaria muralis G. Gaertn., B. Mey. and Scherb	5	E, C
Fumaria capreolata L.	3	E, N
Gamochaeta americana (Mill.) Wedd.	5	S, C, W
Gnaphalium cheiranthifolium Mill.	3	N, S, E
Gnaphalium spicatum Mill.	5	N, E
Gnaphalium polycephalum Mill.	6	C, S
Gnaphalium sp.	1	C
Holcus lanatus L.	4	C, N
Hypochaeris radicata L.	3	S, W, N
Lepidium bipinnatifidum Desv.	7	E, S, C, W, N
Lolium multiflorum Lam.	5	W, C
Megathyrsus maximus (Jacq.) B.K. Simon and S.W.L. Jacobs	2	E
Myosotis sylvatica Ehrh. ex Hoffm.	2	E
Oxalis corniculata L.	4	W, N
Oxalis jacquiniana Kunth.	3	N
Oxalis sp.	3	W
Plantago major L.	3	S, N
Poa annua L.	3	C
Polygonum nepalense Hort. Elden. ex Fenz	1	C
Rumex crispus L.	1	C
Senecio madagascariensis (Humbert) H. Jacob.	5	C, W
Senecio vulgaris L.	10	E, S, W
Sonchus asper (L.) Hill	1	O
Sonchus oleraceus L.	5	E, S, C, N
Spergula arvensis L.	1	E
Taraxacum officinale F.H. Wigg.	14	E, S, W, C
Thlaspi arvense L.	1	E
Trifolium pratense L.	6	W, N
Trifolium repens L.	2	C, W

The letters indicate the approximate location in Bogotá in which the samples were collected. C= center, E = east, N= north, S= south, W= west.
